# A novel continuous hydrodynamic cavitation technology for the inactivation of pathogens in milk

**DOI:** 10.1016/j.ultsonch.2020.105382

**Published:** 2020-11-13

**Authors:** Xun Sun, Xiaoxu Xuan, Li Ji, Songying Chen, Jingting Liu, Shan Zhao, Seulgi Park, Joon Yong Yoon, Ae Son Om

**Affiliations:** a Key Laboratory of High Efficiency and Clean Mechanical Manufacture, Ministry of Education, School of Mechanical Engineering, Shandong University, Jinan 250061, China; b National Demonstration Center for Experimental Mechanical Engineering Education, Shandong University, Jinan 250061, China; c Shandong Key Laboratory of Water Pollution Control and Resource Reuse, School of Environmental Science and Engineering, Shandong University, Qingdao 266237, China; d Department of Food and Nutrition, Hanyang University, Seoul 04763, Republic of Korea; e Department of Mechanical Engineering, Hanyang University, Ansan 15588, Republic of Korea

**Keywords:** Milk treatment, Continuous hydrodynamic cavitation, Thermal characteristics, Bacterial inactivation, Nutritional composition, Safety

## Abstract

Hydrodynamic cavitation is a powerful tool for the enhancement of various processing applications. This study utilizes continuous hydrodynamic cavitation (CHC) for the inactivation of pathogens in milk for the first time. The thermal characteristics, inactivation performance, damage on the nutritional composition, product safety, and cost of the advanced rotational hydrodynamic cavitation reactor at pilot scale were comprehensively investigated. The inactivation results demonstrated that 5.89, 5.53, and 2.99 ± 0.08 log reductions of *Escherichia coli*, *Staphylococcus aureus*, and *Bacillus cereus* were achieved, respectively, at a final treatment temperature of 70 °C for 1–2 s. Moreover, the detrimental effect of CHC on the nutritional composition of milk, including mineral, fat, protein, and vitamin contents, was similar to that of high-temperature short-time method. The change in the concentrations of general bacteria and *E. coli*, as well as the pH value and acidity of the CHC treated milk stored at 5 °C for 14 days was found to be close to that of low-temperature long-time pasteurized milk. The cost of the present CHC treatment was $0.00268/L with a production rate of 4.2 L/min. CHC appears to be a remarkable method for the continuous processing of milk, as well as other liquid foods with high nutrition and “fresh-picked” flavor, due to its high efficacy, good scalability, high production capacity, and low operating and equipment costs.

## Highlights

• CHC is presented as a new method for the inactivation of pathogens in milk.
• CHC treatment is more effective than HTST.
• The effect of CHC on milk nutrition is similar to that of HTST.
• The safety of CHC milk is similar to LTLT and lower than that of HTST.
• CHC treatment costs $0.00268/L at a production rate of 4.2 L/min.

## Graphical abstract

**Figure:**
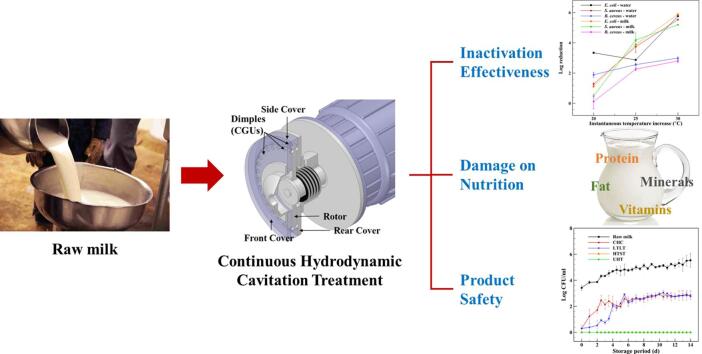

## 1 Introduction

Dairy products, one of the five main food groups of a healthy balanced diet, are widely considered to be nutrient-dense and health-promoting foods [1]. They offer not only macro- and micronutrients (e.g., carbohydrates (lactose), fatty acids, proteins, vitamins, and minerals), but also bioactive peptides, milk fat globule membrane, prebiotics, and probiotics [2]. Due to population growth, rising incomes, and health consciousness, the dairy industry has been expanding rapidly all over the world. In 2018, the global dairy market reached a value of US$673.8 billion and is expected to increase to US$1032.7 billion by 2024 [3]. In dairy processing, pasteurization or sterilization is the most important stage, whereby the pathogenic and spoilage bacteria in the raw milk are eliminated to ensure the safety and extend the storage life of dairy products. Continuous thermal treatments, such as high-temperature short-time (HTST) and ultra-high-temperature (UHT) treatments, are widely utilized around the world [4]. Heating can, to a certain extent, alter the sensory characteristics (e.g., appearance, color, flavor, and texture) and nutritional value of milk, which is determined by the treatment temperature and duration [5]. Therefore, to satisfy the growing demand for healthier dairy products, researchers have been optimizing the equipment and processing parameters of conventional thermal treatments to improve the product quality and economic efficiency. Meanwhile, the food industry is constantly searching for emergent mild processing technologies, such as ultraviolet (UV) irradiation [6], high-pressure processing [7], plasma [8], light pulse [9], pulsed electric field (PEF) [10], and high hydrostatic pressure [11], to obtain high-quality food with “fresh-like” characteristics [12]. Recently, cavitation was found to be an effective means for processing milk as well as other liquid foods [13].

Cavitation is a phenomenon in which a rapid phase-change occurs in a liquid over an extremely short time period, causing existing cavitation nuclei to grow and collapse driven by external input energies [14]. The huge energy involved, including mechanical (shock waves, microjets, and shear stresses), thermal (local hotspots), and chemical effects (hydroxyl radicals), is released into the surrounding liquid during bubble collapse [15], leading to highly destructive effects on various microorganisms [16], [17]. Cavitation can be effectively induced by either ultrasound (acoustic cavitation, AC) or a local pressure drop (hydrodynamic cavitation, HC) [18].

Research and development of AC in the food industry have been ongoing for many years [19]. Compared with conventional thermal treatments, AC can improve the microbiological and physicochemical qualities of various liquid foods, e.g., milk [20] and fruit [21] and vegetable juices [22], as well as preserving the bioactive compounds and nutritional quality [13]. However, due to the characteristics of AC generation, scale-up and energy efficiency are the two major issues hindering industrial-scale treatment by AC [23], [24]. On the other hand, HC has recently been shown to have great potential for industrial applications for various liquid foods such as yogurt [25], skim [26] and peanut milks [27], beer [28], and apple [29] and tomato juices [30], through mechanisms similar to those of AC [31]. Compared with AC reactors (ACRs), HC reactors (HCRs) have the advantages of a simple structure, good scalability, and low equipment and operating costs [32].

Due to the unsatisfactory scalability of ACRs, and the low cavitation intensity generated by conventional HCRs (e.g., Venturi and orifice types), most research on cavitation pasteurization or sterilization has focused only on batch processing which is not suitable for industrial-scale applications. Recently, advanced rotational HCRs (ARHCRs) with different mechanisms of cavitation generation to conventional HCRs have shown treatment efficacy and economic benefits far beyond those of traditional devices for various applications [33], [34], e.g., removal of microorganisms [35], [36], waste-activated sludge (WAS) treatment [37], [38], [39], [40], organic wastewater treatment [41], [42], biofuel synthesis [43], [44], delignification [45], fibrillation [46], and intensification of biogas production [47]. The advent of ARHCRs allows for the continuous HC (CHC) treatment of liquid foods.

To the best of our knowledge, no research on CHC treatment for liquid foods has been reported to date. Based on our previous study of the thermal [48] and disinfection [49] performance of a representative AHRCR, here we present a comprehensive investigation on a new continuous method for the inactivation of pathogens in milk utilizing CHC. The thermal characteristics of the AHRCR were analyzed. The effects of CHC on the microbial inactivation, nutritional composition, and safety of milk were evaluated. Finally, the possible mechanism and application perspective of CHC were discussed.

## 2 Materials and methods

### 2.1 ARHCR design and experimental setup

**Fig. 1:**
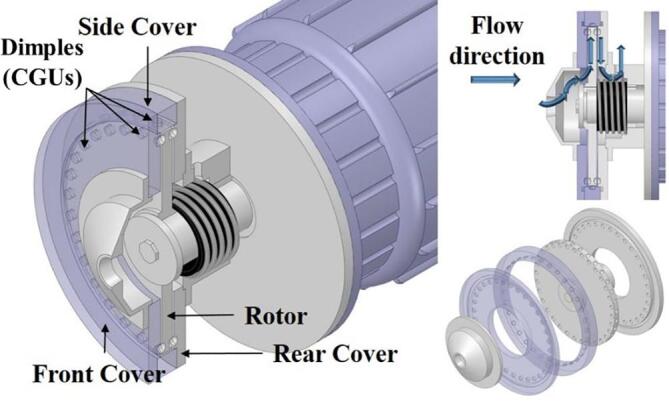
Schematic diagram of the ARHCR [50].

**Fig. 2:**
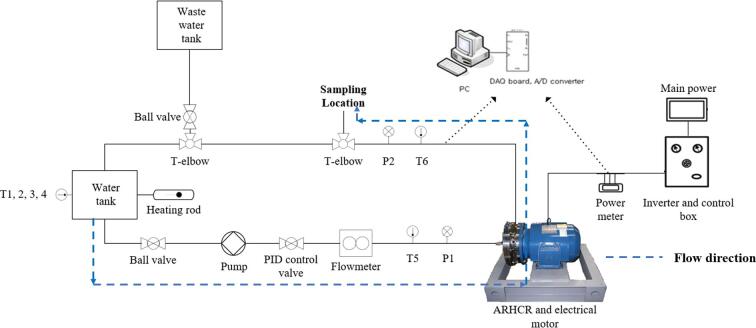
Schematic diagram of the experimental setup of the ARHCR.

To artificially generate HC, a novel ARHCR with a stator-rotor interaction structure was developed, as shown in Fig. 1 [50]. The device included a front cover, side cover, rear cover, and a rotor driven by an electrical motor. Thirty-two cone-cylinder-shaped cavitation generation units (CGUs) were processed on the surface of the rotor, front cover, and rear cover, respectively, equally spaced in a single row. The distance between each cover and the rotor was 1 mm. Fig. 2 illustrates a schematic of the open-loop experimental setup for ARHCR testing. To achieve a continuous treatment mode, the liquid treated by the ARHCR was drained directly to the wastewater tank. The rotor was driven by a 15 kW, three-phase 380 V electrical motor with a standard efficiency of 90.2%. The power consumption of the motor was measured by a clamp-on power meter. The rotational speed was controlled by a power inverter. The flow rate was adjusted and measured utilizing a proportional-integral-derivative valve and an electrical flowmeter. The temperatures of the upstream and downstream flows of the ARHCR were measured by a pair of PT100 ohm sensors. Specifics of the ARHCR and experimental setup have been published and discussed comprehensively elsewhere [50] and thus are not described in detail here.

The uncertainties of the obtained data were calculated using Type B evaluation of uncertainty according to the *Guide to the expression of uncertainty in measurement* [51] from the International Standards Organization. Standard uncertainties are given in Table S1 in the Supplementary Material, calculated using the accuracies of the measurement equipment and the maximum measured or calculated values. As the standard uncertainties are negligible compared with the maximum values, the measured and calculated results of the experiment are considered to be highly reliable.

### 2.2 Test microorganisms

Three representative bacterial strains, *Escherichia coli* (*E. coli*) (ATCC 8739), *Staphylococcus aureus* (*S. aureus*) (ATCC 6538), and *Bacillus cereus* (*B. cereus*) (ATCC 14579), were utilized as model bacteria to evaluate the inactivation performance of the ARHCR. The strains were obtained from the Korean Culture Center of Microorganisms. Three subcultures of each bacterial species were inoculated in tryptic soy broth (Difco Laboratories, Division of Becton Dickinson and Co., Sparks, MD, U.S.A.) and cultured for 24 h at the appropriate temperature (37 °C for *E. coli* and *S. aureus*, 30 °C for *B. cereus*). Before the CHC treatment, the cultured bacterial species were inoculated in distilled water or UHT milk which was purchased from local market.

After CHC treatment, the bacteria were inoculated on 3M^TM^ Petrifilm plates (3 M Co., St. Paul, MN, U.S.A.) in accordance with the Association of Official Analytical Chemists Official Method 991.14 and incubated for 24 ± 4 h at the appropriate temperature (37 °C for *E. coli* and *S. aureus*, 30 °C for *B. cereus*). The concentrations of the bacterial solutions were measured using a colony counter and recorded as colony-forming units per mL (CFU/mL) from three independent experiments. Microbial analysis adhered to the *Korean Food Standards Code 2018* (*KFSC 2018*) recommend by Ministry of Food and Drug Safety, Republic of Korea.

### 2.3 Fundamental parameters

Three fundamental parameters were utilized to evaluate the thermal performance of the ARHCR: instantaneous temperature increase (ITI), heat generation rate (HGR), and thermal efficiency (TE) [48].

The ITI (ΔT, °C) of the liquid flowing through the ARHCR can be defined as follows:

$$\Delta T=T_{\mathrm{out}}-T_{\mathrm{in}},$$

where $T_{\mathrm{out}}$ and $T_{\mathrm{in}}$ are the outlet and inlet temperatures, respectively, in °C.

The HGR ($\dot{H}$, MJ/h) produced by the ARHCR is defined as:

$$\dot{H}=\rho_{\mathrm{out}}QC_{p}\Delta T,$$

where Q is the flow rate (m^3^/h), $C_{p}$ is the specific heat of water (4.1868 kJ/kg K), and $\rho_{\mathrm{out}}$ is the outlet fluid density (kg/m^3^), defined as

$$\rho_{\mathrm{out}}=1000-0.0178\times {\left|T_{\mathrm{out}}-4\right|}^{1.7}.$$

The TE ($\eta_{t}$, %) of the ARHCR is defined as

$$\eta_{t}=\frac{\dot{H}}{{\dot{E}}_{m}\times 3.6}\times 100\%,$$

where ${\dot{E}}_{m}$ is the measured electrical consumption rate of the motor in kW.

### 2.4 Experimental procedures

As the treatment temperature is crucial to the inactivation performance of CHC, the thermal performance of the ARHCR was evaluated by 26 tests (from 4.04 to 16.88 L/min) at a rotational speed of 3600 rpm and a pump pressure of 0.5 bar. The shaft power and the temperatures and flow rates of the inlet and outlet fluid were recorded every second for 120 s under steady operating conditions. The mean value of each variable over the 120 s measurement period was calculated.

To evaluate the inactivation performance, for each set, 80 L of distilled water or UHT milk, inoculated with *E. coli*, *S. aureus*, or *B. cereus* at a concentration of 10^5^ CFU/mL, was utilized as the simulated effluent. The effluents were independently treated by the ARHCR under three operating conditions, as shown in Table 1 (cases 1 to 18). The pump pressure and rotational speed were set at 0.5 bar and 3600 rpm, respectively. The liquid in the tank was pre-heated to a temperature below the point at which bacterial inactivation would occur by adding a heating rod. When the desired temperature was obtained, the liquid was inoculated with the bacterial culture and was adequately mixed. To determine the bacterial concentration, two 100 mL aliquots were taken before and after the CHC treatment for each case.

**Table 1:** Treatment conditions to assess the performance of the ARHCR for the inactivation of *E. coli*, *S. aureus*, and *B. cereus* in water and milk.

Case	Treatment condition				Fluid	Bacterium
	*T_in_* (℃)	*T_out_* (℃)	*ΔT* (℃)	*Q* (L/min)		
1	40	60	20	7	Water	*E. coli*
2						*S. aureus*
3						*B. cereus*
4					Milk^a^	*E. coli*
5						*S. aureus*
6						*B. cereus*
7	40	70	30	4.2	Water	*E. coli*
8						*S. aureus*
9						*B. cereus*
10					Milk^a^	*E. coli*
11						*S. aureus*
12						*B. cereus*
13	45	70	25	5.5	Water	*E. coli*
14						*S. aureus*
15						*B. cereus*
16					Milk^a^	*E. coli*
17						*S. aureus*
18						*B. cereus*
19	40	70	30	4.2	Raw milk	N/A

a Bacteria-inoculated UHT sterilized milk.

The effect of CHC treatment on the nutritional composition of milk was assessed by comparing the mineral, fat, protein, and vitamin contents before and after treatment (case 19). To evaluate milk safety, the change in the concentrations of general bacteria and *E. coli*, as well as the pH value and acidity, of the CHC treated milk during the storage at 5 °C for 14 days was compared with that of raw, long-time low-temperature (LTLT), HTST, and UHT milks.

### 2.5 Analysis methods

#### 2.5.1 Minerals

The analysis of the minerals in milk, including Ca, Zn, P, Mg, and K, was conducted in accordance with the *KFSC 2018*. A microwave digestion system (Ethos 1, Milestone, USA) was utilized to prepare the samples for the analysis which was carried out by inductively coupled plasma (ICP, Jobin Yvon JY 38 Plus, Longjumeau, France). The operating conditions of the ICP are shown in Table S2.

#### 2.5.2 Fat and protein

Fat content was analyzed by the Gerber method as recommended in the *KFSC 2018*. Briefly, samples were mixed with sulfuric acid and amyl alcohol and placed in warm water. The fat layer was extracted following centrifugation of over 700 rpm. Protein content was determined according to the semi-micro Kjeldahl method as recommended in the *KFSC 2018*.

#### 2.5.3 Vitamins

In accordance with the *KFSC 2018*, vitamin contents, including vitamins C, B_2_, B_12_, D, and A, were determined by HPLC (Nanospace SI-1, Shiseido, Japan) equipped with a UV detector (UV 3200, Shiseido, Japan). The conditions utilized for the analysis of fat-soluble and water-soluble vitamins are shown in Tables S3 and S4, respectively.

#### 2.5.4 pH and acidity

In accordance with the *KFSC 2018*, pH value was measured with a pH meter. Titratable acidity was used as a measure of milk acidity. The acidity percentage was determined by the following equation:

(5) $$\mathrm{Acidity}\;\left(\%\right)=0.1\;N\;NaOH\;titration\left(\mathrm{mL}\right)/10$$

## 3 Results and discussion

### 3.1 Thermal characteristics

The most directly observable effect of HC is the heating of liquids at the macro level. Thermal characteristics are therefore an important and intuitive index for evaluating the cavitation intensity of HCRs. Furthermore, it has been confirmed that proper treatment temperature is a positive contributing factor to the effectiveness of HC, such as for the degradation of organic matter [52], [53], WAS treatment [54], [55], and disinfection [30], [56]. More importantly, thermal characteristics proved to be the dominant factor in the efficacy of the CHC process in the present study. Therefore, thermal characteristics have to be studied in detail.

**Fig. 3:**
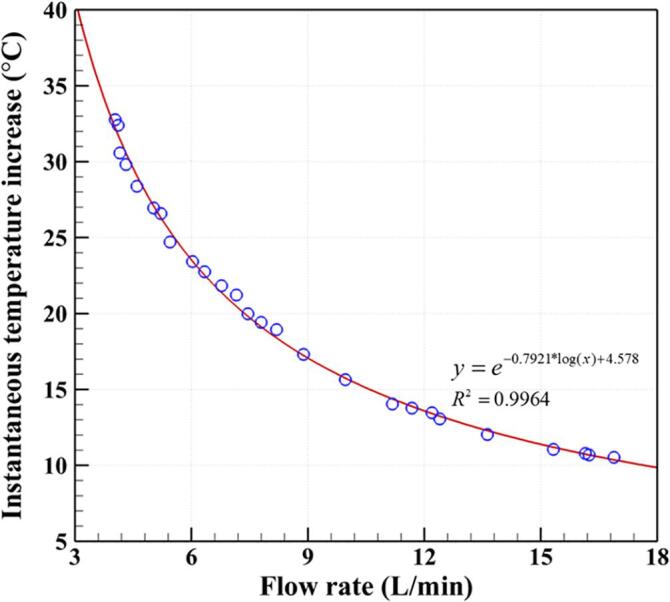
Effect of the flow rate on the instantaneous temperature increase at a rotational speed of 3600 rpm and a pump pressure of 0.5 bar.

Fig. 3 presents the effect of flow rate on the ITI for a rotational speed of 3600 rpm and a pump pressure of 0.5 bar. Decreasing the flow rate from 16.88 to 4.04 L/min results in an increase in ITI from 10.53 to 32.78 °C. This trend becomes more obvious at flow rates below 6 L/min. For the same rotational speed, the heating effect generated by the ARHCR is “concentrated” at lower flow rates, causing a stronger heating effect as a result of the cavitation, leading to a higher ITI, and vice versa [48]. Even though higher ITIs can be beneficial for CHC treatment, which will be described in the next section, it was difficult to decrease the flow rate below 4.04 L/min to achieve higher ITIs. This is because the rotor may become unstable and rub against the covers. This was evidenced by an abnormal noise that was distinct from that heard under normal conditions, which was produced at excessively low flow rates. Such operating conditions can rapidly degrade the durability of the reactor (especially the seal) and eventually cause rotor malfunctions, such as a sudden rotor jam. This may be caused by insufficient fluid passing through the reactor to fill the clearances between the rotor and covers. The maximum ITI of the ARHCR was found to be 30 °C under steady operating conditions.

**Fig. 4:**
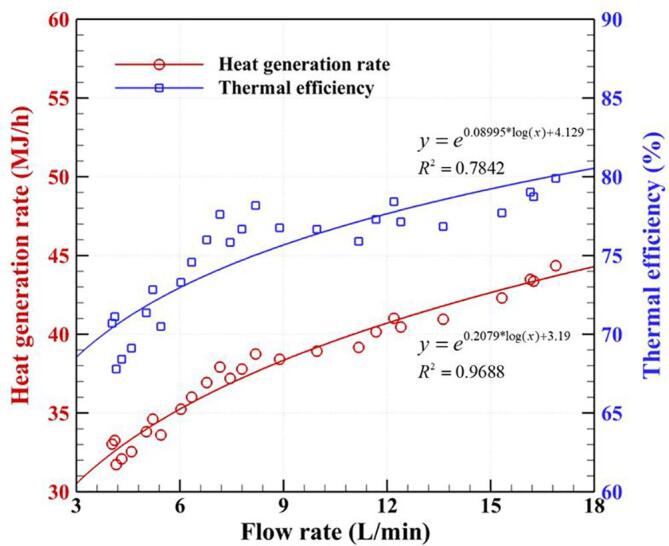
Effect of the flow rate on the heat generation rate and thermal efficiency at a rotational speed of 3600 rpm and a pump pressure of 0.5 bar.

Fig. 4 illustrates the effect of flow rate on the HGR and TE for a rotational speed of 3600 rpm and a pump pressure of 0.5 bar. The curves show a high degree of similarity in the relationship between flow rate and each of the two quantities. A decrease in the flow rate contributes to a decrease in both HGR (from 44.36 to 33.03 MJ/h) and TE (from 79.89 to 70.70%), which is similar to the results found in our previous study [48], [57]. At higher flow rates, the large volume of incoming liquid provides enough nuclei for the cavitation generation process, however, it also immediately distributes a large amount of the released thermal energy. Moreover, due to the disadvantages of low flow rates mentioned above, low flow rates have a negative influence on the thermal performance of the ARHCR.

### 3.2 Inactivation performance

*E. coli* is an important indicator of safety in water and foods and is present in human and animal feces. *S. aureus.* and *B. cereus* are pathogenic microorganisms that can be present in raw milk [58]. Therefore, to evaluate the inactivation performance of CHC, different conditions of inlet temperature, outlet temperature, and ITI for the removal of *E. coli*, *S. aureus*, and *B. cereus* from water and UHT milk were examined. In our experience, the ITI and outlet temperature are crucial to the inactivation effectiveness. To achieve satisfactory results, the inlet temperature was set to either 40 or 45 °C, which would not cause inactivation of the bacteria [49]. The corresponding inactivation results are shown in Table 2.

**Table 2:** Effects of different treatment conditions of CHC on the concentrations of *E. coli*, *S. aureus*, and *B. cereus*.

*T_in_* (℃)	*T_out_* (℃)	*ΔT* (℃)	*Q* (L/min)	Fluid	Bacterium	Initial concentration (CFU/mL)	Treated concentration (CFU/mL)	Log reduction
40	60	20	7	Water	*E. coli*	5.67 ± 0.17^a^	2.23 ± 0.16	3.34 ± 0.01
					*S. aureus*	5.41 ± 0.04	4.14 ± 0.17	1.27 ± 0.08
					*B. cereus*	5.43 ± 0.25	3.54 ± 0.09	1.89 ± 0.16
				Milk^b^	*E. coli*	5.58 ± 0.07	4.47 ± 0.30	1.11 ± 0.05
					*S. aureus*	4.03 ± 0.11	3.55 ± 0.08	0.48 ± 0.12
					*B. cereus*	5.66 ± 0.57	5.54 ± 0.09	0.12 ± 0.48
40	70	30	4.2	Water	*E. coli*	5.76 ± 0.07	ND^c^	5.76
					*S. aureus*	5.53 ± 0.16	ND	5.53
					*B. cereus*	5.80 ± 0.05	2.81 ± 0.04	2.99 ± 0.08
				Milk^b^	*E. coli*	5.89 ± 0.05	ND	5.89
					*S. aureus*	5.19 ± 0.06	ND	5.19
					*B. cereus*	5.92 ± 0.08	3.12 ± 0.07	2.80 ± 0.11
45	70	25	5.5	Water	*E. coli*	5.07 ± 0.08	2.20 ± 0.07	2.87 ± 0.05
					*S. aureus*	5.52 ± 0.03	1.78 ± 0.35	3.74 ± 0.37
					*B. cereus*	5.23 ± 0.07	2.67 ± 0.06	2.56 ± 0.09
				Milk^b^	*E. coli*	4.56 ± 0.03	0.69 ± 0.4	3.87 ± 0.08
					*S. aureus*	5.36 ± 0.04	1.18 ± 0.52	4.18 ± 0.49
					*B. cereus*	5.82 ± 0.03	3.56 ± 0.07	2.26 ± 0.09

a The value is expressed as mean ± standard deviation (n = 3).

b Bacteria-inoculated UHT sterilized milk.

c Not detected.

**Fig. 5:**
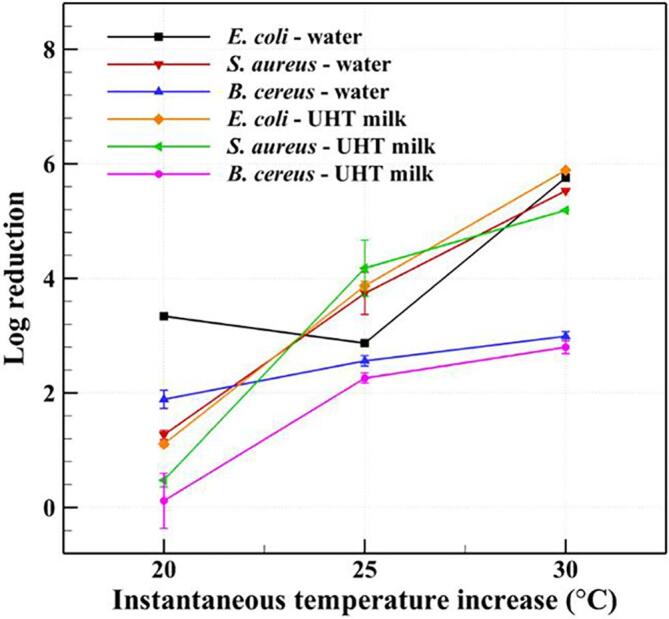
Effect of the instantaneous temperature increase on the log reductions of *E. coli*, *S. aureus*, and *B. cereus*.

CHC treatment was found to be highly effective for inactivating bacteria. Specifically, for the cases of *ΔT* = 20 °C and *T_out_* = 60 °C, CHC only results in 0.12 ± 0.48 to 3.34 ± 0.01 log reduction of the three bacterial species in water and UHT milk. However, when *ΔT* is increased to 30 °C (*T_out_* = 70 °C), the log reductions of *E. coli*, *S. aureus*, and *B. cereus* obtained by CHC were increased by approximately 1.72, 4.35, and 1.58 times in water and 5.31, 10.81, and 23.3 times in UHT milk, respectively. The *E. coli* and *S. aureus* in both water and UHT milk are reduced to undetectable levels under these conditions. Moreover, when the ITI and outlet temperature are specified as 25 and 70 °C, respectively, the inactivation effects for all bacteria are seen to deteriorate somewhat compared with those observed for an ITI of 30 °C. This indicates that, despite the outlet temperatures being identical, an increase of only 5 °C in the ITI can lead to significant improvements in the effectiveness of CHC. Fig. 5 is a graphical representation of the results given in Table 2, showing the effect of ITI on the log reduction of the three species of bacteria, which presents the importance of ITI in a more intuitive manner.

In general, HTST (71–75 °C, 15–40 s) is able to achieve a 6-log reduction of pathogenic microorganisms such as *E. coli* and *S. aureus*; CHC treatment completely eliminated these microorganisms, equivalent to similar log reductions, at a similar treatment temperature (70 °C) for an even shorter duration (1–2 s). On the other hand, *B. cereus*, a spore-forming bacterium, is highly resistant to high temperatures, cleaning, and disinfection agents [59] and the effect of HTST on *B. cereus* is limited [60]. For instance, Stadhouders, et al. [61] reported that milk must be treated at 125 °C for 10–20 s to inactivate all *B. cereus* spores. The present CHC treatment achieved a maximum of 2.99 log reduction for *B. cereus* (corresponding to an elimination rate of 99.85%), while a similar level of inactivation can be obtained by conventional heating treatment at 100 °C for 4 min (99.95%), 110 °C for 60 s (99.93%), or at 120 °C for 10 s (99.87%) [62]. This demonstrates that CHC is highly destructive for these pathogenic microorganisms and is able to eliminate them more effectively under conditions of lower temperatures and shorter durations, than conventional heating treatments such as HTST or UHT.

It should be noticed that the CHC treatment may only kill the vegetative cells of *B. cereus*, the spores may still remain in the treated water or milk. The formation of a spore from a vegetative *B. cereus* cell starts when it suffers from unfavorable external environment, e.g., CHC treatment. First, an asymmetrically positioned septum forms and splits the cell into a smaller and a bigger part by the asymmetrical division of the chromatin. Then, the smaller part (i.e., fore spore) is engulfed by the mother cell, a thick layer of peptidoglycan around the chromatin network is formed (i.e., the cortex). Finally, the mature spore is freed from the mother cell by lysis [63]. The generated dormant spores are highly resistant to various extreme conditions, e.g., low pH, high temperature, antibiotic, UV, or gamma radiation [64]. The spores can dormant and germinate to form vegetative cells when encountering favorable conditions, causing two foodborne illnesses in humans namely, diarrhea and emesis [65]. Although CHC treatment, like HTST, cannot remove all spores of *B. cereus*, immediate cooling and cold storage after the treatment (e.g., 5 °C [66]) can prevent activating them.

The results also prove that treatment temperature dominates microbial inactivation. The effect of treatment temperature on microorganisms has been ignored, with few published studies assessing this variable. For instance, our previous study [49] reported that the concentration of *E. coli* was reduced only after temperatures exceeded approximately 54 °C when treated by the same ARHCR as the present study, and the disinfection rates of 100% can be achieved at approximately 65 °C. More direct research on the temperature of HC treatment was proposed by Mane, et al. [56], who found that the disinfection rate of *S. aureus* achieved by a vortex diode increased from 33.5 to 69.8% by increasing the treatment temperature from 28 to 50 °C. The possible mechanism of CHC treatment will be discussed in section 3.5.

### 3.3 Nutritional composition

To evaluate the effect of CHC treatment on the nutritional value of milk, the contents of various minerals (Ca, Zn, P, Mg, and K), milk fat, milk protein, and vitamins (vitamins C, B_2_, B_12_, D, and A) in raw and CHC treated milks were determined, and are presented in Table 3. The effect of CHC was compared with that of HTST treatment reported in the literature. On the whole, the damage caused by CHC to milk nutrition is similar to that of HTST.

**Table 3:** Effect of CHC treatment on the nutritional composition of milk.

Nutritional composition		Unit	Content		Loss	
			Initial	Final	CHC	HTST
Mineral	Ca	mg/100 g	115.55 ± 0.21^a^	106.74 ± 0.23	7.62%	<10% [5]
	Zn		0.38 ± 0.04	0.30 ± 0.01	21.05%	
	P		86.97 ± 0.16	84.88 ± 0.14	2.40%	
	Mg		11.11 ± 0.28	9.81 ± 0.03	11.70%	
	K		125.63 ± 0.18	123.12 ± 0.02	2.00%	
Milk fat		%	3.1 ± 0.02	3.03 ± 0.04	0.07%^b^	0.03%^b^[72]
Milk protein			3.15 ± 0.03	2.68 ± 0.09	0.47%^b^	0.43%^b^[75]
Vitamin	C	mg/100 g	3.1 ± 0.03	2.7 ± 0.10	12.90%	16.6% [76]
	B_2_		0.12 ± 0.01	0.1 ± 0.01	9.67%	<5% [77]
	B_12_	μg/100 g	0.59 ± 0.14	0.47 ± 0.07	20.34%	11.6–16.7% [78]
	D		0	0	N/A	–
	A	μg RE/100 g	29.03 ± 0.19	23.01 ± 0.25	20.74%	23% [79]

a The value is expressed as mean ± standard deviation (n = 3).

b The value was calculated by subtracting the final content (%) from the initial content (%).

Milk is an important source of minerals, such as Ca, P, Zn, Mg, and K, that are essential for skeletal development, protein synthesis, muscle contraction, nerve function, or fluid balance of the human body [67]. It has been found that HTST has a limited negative effect on the mineral content of milk (less than 10% reduction), because the transformation of minerals from the soluble to colloidal state is partially or completely reversible after heating, during the stages of cooling and storage [68]. Compared with raw milk, the contents of Ca, P, Mg, and K in the CHC treated milk are reduced by 7.62%, 2.40%, 11.70%, and 2.00%, respectively, while the loss of Zn reaches 21.05%. Therefore, there is no significant difference in the mineral contents of milk after CHC treatment. Even though soluble minerals such as Ca and P can be converted to insoluble forms during the treatment, the total content remains unchanged. These insoluble contents can resolubilize within 24 to 48 h when milk is refrigerated at 5 °C, thereby restoring the mineral content to the original level and having little effect on the nutritional value of milk [69].

Milk fat, which is an important source of the bioactive fatty acid conjugated linoleic acid in the human diet [70], plays an important role in forming the smell and taste of dairy products. It exists mainly in globules surrounded by a complex membrane consisting of a mixture of unsaturated phospholipids, proteins, glycoproteins, and other minor constituents [71]. It is widely known that heating processes can damage the flavor of dairy products by forming lactones or methyl ketones, affecting the product quality. The amount of milk fat lost during CHC treatment (0.07%) is similar to that observed in HTST (0.03%) [72]. Nevertheless, the specific lipid profiles can be changed by the destructive effects of HC, which was confirmed by Gregersen, et al. [73]. They found that the volume-based average milk fat globule (MFG) size was reduced from 4.09 μm for an untreated sample to 3.29–1.40 μm for HC-treated samples. The reduction in MFG can be attributed to high pressure, shear force, and temperature during bubble collapse. In addition, the mechanical and thermal effects can also change the proteins associated with MFG membrane (MFGM) [74]. Gregersen, et al. [73] also found that the HC treatment led to a decrease in MFGM proteins (from 88.7 ± 1.1 to 70.2 ± 1.8%) and the increases in caseins (from 2.2 ± 2.2 to 4.1 ± 1.2%) and whey protein (from 7.4 ± 0.8 to 21.3 ± 1.7%). It should be noticed that MFGM can be oxidized by the hydroxyl radicals with an oxidation potential of 2.8 V generated during HC treatment. As the focal point for the oxidation of milk lipids, the oxidation of MFGM can result in generalized oxidation of milk fat triglycerides, and consequently, give rise to undesirable volatile flavor compounds, potentially toxic oxidation products, and a general deterioration in the quality of the fat [71]. The effect of CHC on the product shelf life by accelerating lipid oxidation reactions has to be further investigated in future.

Milk protein is used in a variety of functional and nutritional applications. The denaturation of these proteins (e.g., whey proteins and enzymes) cannot be avoided during the heating process. Among the whey proteins, β-1 g, whey albumin, and immunoglobulins denature at 60 °C or higher. In general, 12–20% and 40–60% of protein can be denatured during LTLT and UHT treatments, respectively [4]. The results of this study indicate that 0.47% of the proteins are damaged by CHC treatment, which is similar to the 0.43% reduction observed after HTST [75].

Milk provides a convenient and economical source of various vitamins that plays vital roles as co-factors for enzymatic reactions in intermediary metabolism or in non-enzymatic physiological functions, such as in the visual process (retinol (vitamin A)), as antioxidants (riboflavin (vitamin B_2_) and ascorbic acid (vitamin C)), and in hematopoiesis (pyridoxine (vitamin B_6_) and cobalamin (vitamin B_12_)) [80]. The contents of vitamins C, B_2_, B_12_, and A are reduced by 12.9%, 9.67%, 20.34%, and 20.74%, respectively, following CHC treatment.

In summary, CHC treatment confers a similar detrimental effect to HTST treatment on the nutritional content of milk. However, heat treatment can influence the allergy protective properties of raw milk [81]. CHC treatment, with lower temperatures and shorter durations, may largely suppress the production of milk allergen compared with HTST.

### 3.4 Safety evaluation

**Fig. 6:**
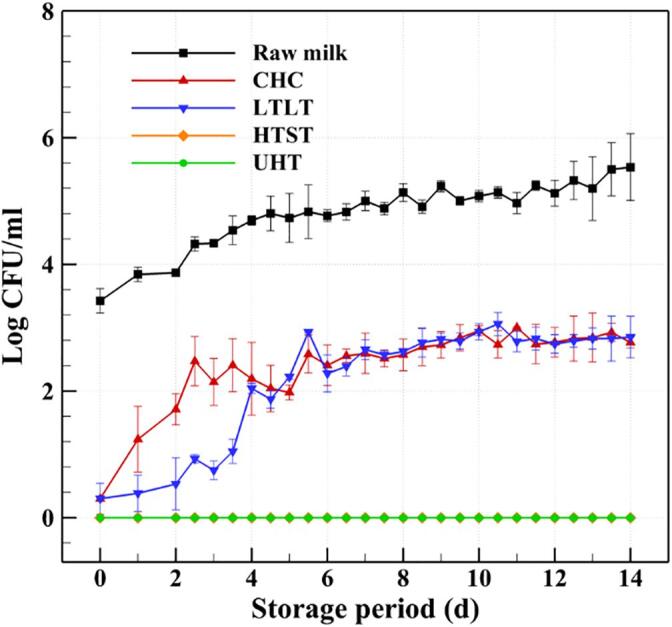
Changes in the concentrations of general bacteria in raw milk and the milks treated by CHC, LTLT, HTST, and UHT stored at 5 °C for 14 days.

**Fig. 7:**
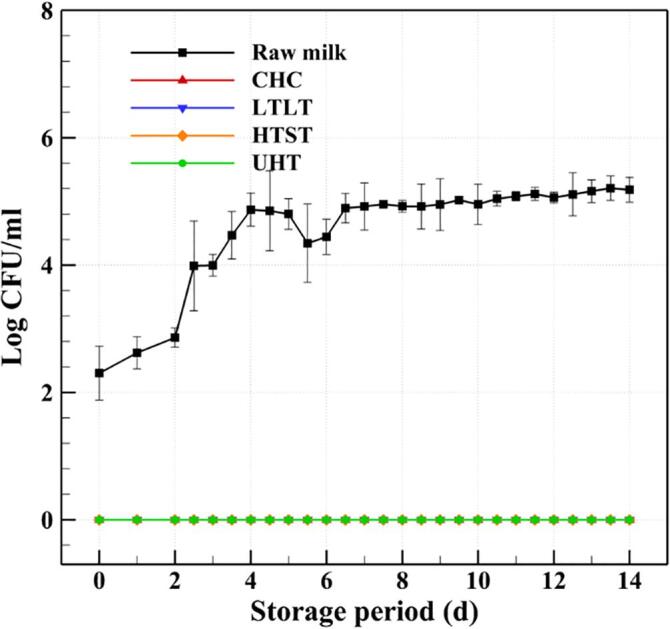
Changes in the concentrations of *E. coli* in raw milk and the milks treated by CHC, LTLT, HTST, and UHT stored at 5 °C for 14 days.

**Fig. 8:**
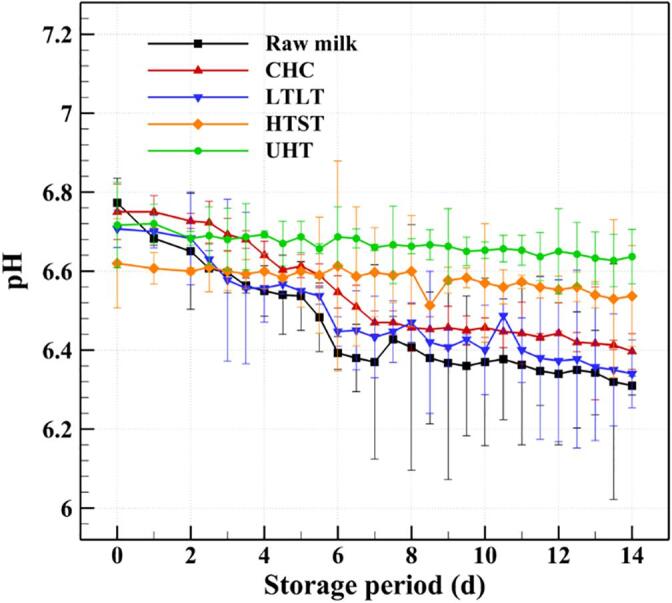
Changes in the pH values of raw milk and the milks treated by CHC, LTLT, HTST, and UHT stored at 5 °C for 14 days.

**Fig. 9:**
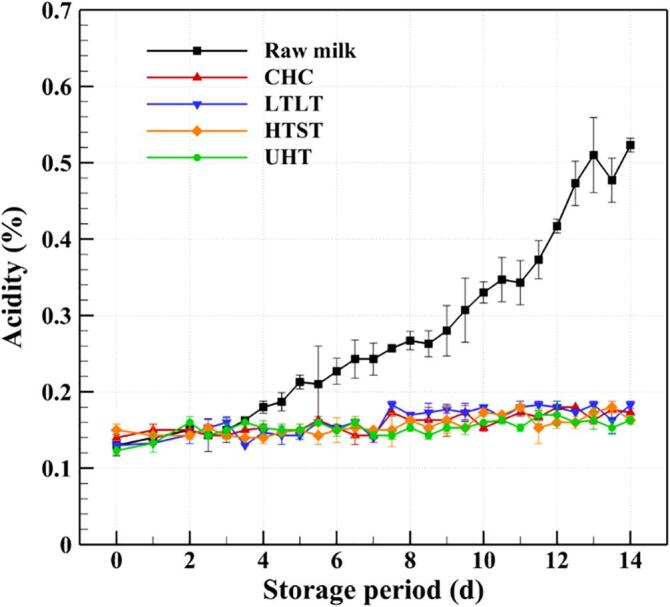
Changes in the acidity values of raw milk and the milks treated by CHC, LTLT, HTST, and UHT stored at 5 °C for 14 days.

In general, milk products are considered safe within the expiration date, depending on the storage temperature, after which microorganisms may grow beyond legal standards and cause the quality of the product to change due to oxidation, hydrolysis, and saccharification [82]. Bacterial spoilage, which is caused by heat-resistant microorganisms or recontamination after pasteurization, is one of the most important factors affecting the safety and storage duration of milk products [83]. The growth and metabolism of bacteria in milk can lead to undesirable changes in aroma and taste, and eventually shorten the shelf life [84]. Therefore, it is vital to evaluate the changes in the biological and chemical properties of treated milk during storage. In the present work, the safety of the CHC treated milk was evaluated by recording the changes in concentrations of *E. coli* and general bacteria, as well as pH and acidity, during storage at 5 °C for 14 days, and comparing these values with those of raw, LTLT, HTST, and UHT milks, as demonstrated in Fig. 6, Fig. 7, Fig. 8, Fig. 9. The specific data in Fig. 6, Fig. 7, Fig. 8, Fig. 9 can be found in Tables S5–8.

Fig. 6 shows that the concentration of general bacteria in the raw milk continuously increases over the storage period, from 3.423 ± 0.193 log CFU/mL on day 0 to 5.533 ± 0.528 log CFU/mL on day 14. The change in the concentration of bacteria in the CHC milk is highly similar to that of the LTLT milk. The bacterial concentration in the CHC milk reaches 2.767 ± 0.086 log CFU/mL on day 14, which is below the 20,000 CFU/mL required by the Pasteurized Milk Ordinance of the United States. However, the HTST and UHT milks show no detectable levels of bacteria or any type of microorganism during storage. Fig. 7 shows that no *E. coli* is detected in any of the treated milks over the 14-day storage period.

A decrease in the pH or an increase in the acidity of milk suggests that the physical properties are becoming unstable due to the growth of microorganisms, which can lead to a defect in quality. Fig. 8 demonstrates that the pH value of CHC milk after storage for 14 days is 6.397 ± 0.045 (reduced from pH 6.750 ± 0.070 on day 0); the corresponding pH values for raw, LTLT, HTST, and UHT milks are pH 6.310 ± 0.024 (reduced from pH 6.773 ± 0.062), pH 6.340 ± 0.086 (reduced from pH 6.707 ± 0.047), pH 6.537 ± 0.128 (reduced from pH 6.620 ± 0.113), and pH 6.637 ± 0.069 (reduced from pH 6.717 ± 0.108), respectively. In addition, the acidities of CHC, LTLT, HTST, and UHT milks on day 14 are 0.173 ± 0.005%, 0.183 ± 0.005%, 0.163 ± 0.005%, and 0.163 ± 0.005%, respectively (Fig. 9). The acidity of CHC milk after storage for 14 days is lower than the legal standard value of 0.18% specified by Korea Food and Drug Administration (2018). Overall, the safety of CHC milk is similar to that of LTLT milk and lower than that of HTST and UHT milks.

### 3.5 Possible bacterial inactivation mechanism

The results of the present study indicate that both the temperature and duration required for bacterial inactivation can be considerably reduced by combining the thermal, physical, and chemical effects induced by HC. For example, with the present CHC treatment, *E. coli* at an initial concentration of ~10^5^ CFU/mL in both water and UHT milk can be completely eliminated at 70 °C with a hydraulic retention time of less than 2 s (the volume of the ARHCR was approximately 0.15 L and the flow rate was 4.2 L/min or 0.07 L/s). By contrast, numerous experiments have shown that heat treatment at 70 °C for 2 min (or equivalent) fails to deliver a 6-decimal reduction in the numbers of *E. coli* [85].

The mechanism by which CHC achieves inactivation is through a combination of mechanical, thermal, and chemical effects. The mechanical effect, resulting from the impact of shock waves with pressures as strong as 6000 GPa [86], micro-jets at a maximum speed of 175 m/s [87], and shear stress as high as 3.5 kPa [88] during bubble collapse, can lead to generalized membrane rupture, causing the loss of cytoplasmic and periplasmic matter from the cell [56], [89]. In some cases, the cell can even be cleaved down the middle [90]. The extreme conditions created during HC can lead to the formation of highly reactive hydroxyl radicals [91], with a standard oxidation potential of 2.8 V [92]. Chain reactions of these radicals may oxidize the sulfhydryl groups and double bonds of important constituents of microorganisms (including membrane surfaces, lipids, and proteins) resulting in irreversible damage to the cells [93]. Moreover, localized hot spots generated by short-lived cavitation bubbles may reach several thousand Kelvin (5200 K [94]) resulting in heating and cooling rates above 10^10^ K/s within microseconds [15], which can lead to irreversible denaturation of membranes, loss of nutrients and ions, ribosome aggregation, DNA strand breaks, inactivation of essential enzymes, and protein coagulation [95].

On the other hand, the combination of the above three effects is also detrimental to the nutritional content. The treatment temperature of CHC (70 °C) is almost identical to that of HTST (71–74 °C), and although the treatment time (1–2 s) of CHC is much shorter than that of HTST (15–40 s), the damage caused by the two methods are similar. This indicates that, in addition to the known thermal effects on nutritional content, the mechanical and chemical effects of CHC also play important roles [21]. The highly reactive unpaired electrons of hydroxyl radicals attack specific chemical bonds of organic nutrients such as proteins and vitamins [96]. In addition, considerably high pressure and shear stress can result in the breakdown of hydrogen and van der Waals bonding [97].

### 3.6 Application perspective

The results indicate that CHC is a promising alternative or complementary technology for milk treatment, with lower temperatures and shorter duration of treatment compared with traditional continuous heating processes. The nutritional content and flavor may also be preserved during this process. For example, Gregersen, et al. [98] found that no irreversible unfolding of whey proteins occurred during HC treatment. In addition, HC can refine the physical properties of dairy products, improving the stability [30], color [27], and antioxidant activity [99], and reducing viscosity [98], which can improve the product quality and please customers.

As well as the quality considerations, the cost in terms of both time and money are important factors when determining the industrial application potential of CHC. The 15 kW pilot-scale ARHCR utilized in the present study provided a production rate (i.e., flow rate) of 4.2 L/min at the optimal operating condition, with an electrical power of 13 kW. Therefore, the treatment cost can be calculated as following: electricity cost ÷ (production rate per hour ÷ electricity required per hour) = $0.052/kWh ÷ (4.2 L/min × 60 min ÷ 13 kWh) = $0.00268/L which is reasonable for a pilot experiment. It should be noted that the cost of preheating stage is not included in this value, as the abundant heat energy generated by the ARHCR (30–45 MJ/h) can be recovered by a heat pump or heat exchanger to preheat the milk before CHC treatment, thereby saving this portion of the operational cost.

Moreover, the ARHCR has reasonable potential for scalability, which was demonstrated in our previous study [48]. When the scale was enlarged from 15 to 55 kW and the rotor diameter increased from 260 to 590 mm, the HGR and TE increased from 48.15 to 200 MJ/h and 82.18 to 90%, respectively. The equipment cost of the ARHCR mainly consists of the raw material (stainless steel) and manufacturing. Without the precise and expensive parts, the price of the ARHCR is much lower than other non-thermal treatment equipment (e.g., the ultrasound horn in AC, UV lamp in UV irradiation, or electrode in PEF), even after scaling. More importantly, after scaling of the ARHCR, the higher thermal performance means a higher ITI can be achieved. Therefore, a wider range of treatment intensities (ITI of 30–70 °C) can be conveniently applied to the raw milk by adjusting the flow rate, depending on the desired product quality and storage duration. The CHC process with a final treatment temperature over 100 °C may destroy all microorganisms including spores and achieved sterilization of liquid foods. The optimizations of CHC processes and devices are needed in future.

## 4 Conclusions

In the present study, a CHC processing method was developed and successfully utilized for the inactivation of pathogens in milk for the first time. Compared with HTST (71–74 °C, 15–40 s), the CHC treatment temperature was lower (70 °C) and treatment duration was shorter (1–2 s). Log reductions of 5.89 (100%), 5.53 (100%), and 2.99 ± 0.08 (99.85%) for *E. coli*, *S. aureus*, and *B. cereus*, respectively, were achieved under optimal conditions, with a production rate of 4.2 L/min and a cost of $0.00268/L. The effect of CHC on the nutritional composition of milk was similar to that of HTST, while the safety characteristics of the CHC milk were similar to LTLT milk and lower than HTST and UHT milks. CHC can be utilized as a promising alternative or complementary technology for producing safe, healthy, and nutritious milk, as well as other liquid food products with “fresh-picked” flavor. Further research on the effects of CHC on the microorganisms, nutrition contents, and safety and the optimizations of CHC devices and processes are needed in future.

## CRediT authorship contribution statement

**Xun Sun:** Investigation, Methodology, Writing - original draft. **Xiaoxu Xuan:** Writing - review & editing, Data curation. **Li Ji:** Writing - review & editing. **Songying Chen:** Writing - review & editing. **Jingting Liu:** Writing - review & editing. **Shan Zhao:** Writing - review & editing. **Seulgi Park:** Investigation. **Joon Yong Yoon:** Conceptualization, Supervision. **Ae Son Om:** Conceptualization, Supervision.

## Declaration of Competing Interest

The authors declare that they have no known competing financial interests or personal relationships that could have appeared to influence the work reported in this paper.
