# Supplementary material for: A novel continuous hydrodynamic cavitation technology for the inactivation of pathogens in milk
Source: Ultrason Sonochem. 2020 Nov 13;71:105382. doi: 10.1016/j.ultsonch.2020.105382 (PMC7786570; doi:10.1016/j.ultsonch.2020.105382)
Supplement: Supplementary data 1 [file mmc1.docx]

**Supplementary Material**

**A Novel Continuous Hydrodynamic Cavitation Technology for the Inactivation of Pathogens in Milk**

Xun Sun ^a,b^, Xiaoxu Xuan ^a,b^, Li Ji ^a,b^, Songying Chen ^a,b^, Jingting Liu ^a,b^,

Shan Zhao ^c^, Seulgi Park ^d^, Joon Yong Yoon ^e*^, and Ae Son Om ^d*^

*^a^* *Key Laboratory of High Efficiency and Clean Mechanical Manufacture, Ministry of Education, School of Mechanical Engineering, Shandong University, Jinan 250061, China*

*^b^ National Demonstration Center for Experimental Mechanical Engineering Education, Shandong University, Jinan 250061, China*

*^c^ Shandong Key Laboratory of Water Pollution Control and Resource Reuse, School of Environmental Science and Engineering, Shandong University, Qingdao 266237, China*

*^d^ Department of Food and Nutrition, Hanyang University, Seoul 04763, Republic of Korea*

*^e^ Department of Mechanical Engineering, Hanyang University, Ansan 15588, Republic of Korea*

**Table S1** Standard uncertainties of measured quantities in the experiment.

| **Measurement quantity** | **Sensor accuracy** | **Measured or calculated maximum value** | **Standard uncertainty** |
| --- | --- | --- | --- |
| Temperature (°C) | ±0.25% | 70.11 | 0.137 |
| Flow rate (L/min) | ±0.5% | 16.88 | 0.149 |
| Shaft power (kW) | ±0.2% | 15.42 | 0.0308 |
| Heat generation rate (MJ/h) | - | 57.21 | 0.112 |
| Thermal efficiency (%) | - | 83.62 | 0.227 |

**Table S2** Operating conditions of the ICP instrument for mineral analysis.

| **Parameter** | | **Setting** |
| --- | --- | --- |
| RF power (W) | | 1,400 |
| Gas flow (L/min) | Nebulizer | 0.8 |
|  | Plasma | 15 |
|  | Auxiliary | 0.2 |
| Wavelength (nm) | | Ca: 315.887 |
|  |  | Mg: 279.079 |
|  |  | Zn: 213.856 |

**Table S3** Operating conditions of the HPLC instrument for fat-soluble vitamin analysis.

| **Classification** | **Vitamin A** | **Vitamin D** |
| --- | --- | --- |
| **Column** | Capcell Pak UG120 C18  (250 mm× 4.6 mm, 5 μm) | Capcell Pak MF C8 SG 80  (150 mm × 4.6 mm, 5 μm)  Capcell Pak C18 UG120V  (35 mm × 2.0 mm, 5 μm)  Capcell Pak C18 UG120V  (250 mm × 1.5 mm, 5 μm) |
| **Mobile phase** | 95% Ethanol | A: Vitamin: Ethanol: Water  (74: 8.3: 17)  B: Coconut: DW (93: 7) |
| **Flow rate** | 0.5 mL/min | Pump 1: 500 μL/min  Pump 2: 100 μL/min |
| **Oven temperature** | 35 ℃ | 40 ℃ |
| **Injection volume** | 5 μL | 40 μL |
| **Detector** | UV detector  (Ex: 340 nm, Em: 460 nm) | UV detector  (254 nm) |

**Table S4** Operating conditions of the HPLC instrument for water-soluble vitamin analysis.

| **Classification** | | **Vitamin B_2_** | | **Vitamin B_12_** | | **Vitamin C** | |
| --- | --- | --- | --- | --- | --- | --- | --- |
| **Column** | Capcell Pak C 18  (UG 80 4.6 mm × 250 mm) | | - Preparation Column  Capcell Pak C8, SG80  (4.6 mm × 150 mm, 5 μm)  - Focusing Column  Capcell Pak C18, UG120V  (2.0 mm × 35 mm, 5 μm)  - Analytical Column  Capcell Pak C18, UG120V  (1.5 mm × 250 mm, 5 μm) | | Shiseido Capcell Pak C18 MG  (4.6 mm × 250 mm, 5 μm) | |  |
| **Mobile phase** | Methanol: 10 mM NaH_2_PO_4_ solution (35: 65) | | A: 5mM KH_2_PO_4_  B: 5 mM KH_2_PO_4_: MeOH  (80: 20) | | 0.05 M KH_2_PO_4_: ACN (98: 2) | |  |
| **Flow rate** | 0.8 mL/min | | A: B = 500: 135 | | 0.55 mL/min | |  |
| **Oven temperature** | 35 ℃ | | 40 ℃ | | 35 ℃ | |  |
| **Injection volume** | 10 μL | | 160 μL | | 5 μL | |  |
| **Detector** | UV detector  (Ex: 445nm, Em: 530 nm) | | PDA  (550 nm) | | UV detector  (254 nm) | |  |

**Table S5** Changes in the concentrations of general bacteria in raw milk and the milks treated by CHC, LTLT, HTST, and UHT stored at 5 ℃ for 14 days.

| **Storage period (d)** | **Raw milk** | **CHC** | **LTLT** | **HTST** | **UHT** |
| --- | --- | --- | --- | --- | --- |
| 0 | 3.423 ± 0.193^a^ | 0.300 ± 0.245 | 0.300 ± 0.245 | 0 | 0 |
| 1 | 3.840 ± 0.114 | 1.237 ± 0.518 | 0.387 ± 0.288 | 0 | 0 |
| 2 | 3.867 ± 0.050 | 1.710 ± 0.246 | 0.533 ± 0.411 | 0 | 0 |
| 2.5 | 4.320 ± 0.112 | 2.470 ± 0.390 | 0.930 ± 0.067 | 0 | 0 |
| 3 | 4.333 ± 0.059 | 2.143 ± 0.369 | 0.747 ± 0.148 | 0 | 0 |
| 3.5 | 4.537 ± 0.225 | 2.407 ± 0.420 | 1.047 ± 0.191 | 0 | 0 |
| 4 | 4.693 ± 0.074 | 2.193 ± 0.574 | 2.043 ± 0.078 | 0 | 0 |
| 4.5 | 4.803 ± 0.274 | 2.040 ± 0.367 | 1.870 ± 0.141 | 0 | 0 |
| 5 | 4.733 ± 0.386 | 1.977 ± 0.117 | 2.223 ± 0.041 | 0 | 0 |
| 5.5 | 4.830 ± 0.422 | 2.583 ± 0.301 | 2.933 ± 0.012 | 0 | 0 |
| 6 | 4.767 ± 0.094 | 2.407 ± 0.323 | 2.277 ± 0.292 | 0 | 0 |
| 6.5 | 4.827 ± 0.128 | 2.557 ± 0.105 | 2.387 ± 0.153 | 0 | 0 |
| 7 | 5.000 ± 0.153 | 2.593 ± 0.318 | 2.653 ± 0.154 | 0 | 0 |
| 7.5 | 4.880 ± 0.099 | 2.513 ± 0.133 | 2.570 ± 0.067 | 0 | 0 |
| 8 | 5.133 ± 0.141 | 2.570 ± 0.250 | 2.623 ± 0.050 | 0 | 0 |
| 8.5 | 4.910 ± 0.104 | 2.697 ± 0.296 | 2.763 ± 0.226 | 0 | 0 |
| 9 | 5.233 ± 0.086 | 2.727 ± 0.208 | 2.817 ± 0.092 | 0 | 0 |
| 9.5 | 5.003 ± 0.068 | 2.840 ± 0.205 | 2.787 ± 0.127 | 0 | 0 |
| 10 | 5.077 ± 0.090 | 2.953 ± 0.078 | 2.933 ± 0.131 | 0 | 0 |
| 10.5 | 5.137 ± 0.091 | 2.733 ± 0.215 | 3.057 ± 0.180 | 0 | 0 |
| 11 | 4.967 ± 0.168 | 3.000 ± 0.014 | 2.780 ± 0.163 | 0 | 0 |
| 11.5 | 5.243 ± 0.076 | 2.740 ± 0.312 | 2.827 ± 0.179 | 0 | 0 |
| 12 | 5.123 ± 0.204 | 2.770 ± 0.232 | 2.743 ± 0.145 | 0 | 0 |
| 12.5 | 5.323 ± 0.301 | 2.827 ± 0.355 | 2.790 ± 0.100 | 0 | 0 |
| 13 | 5.197 ± 0.503 | 2.843 ± 0.386 | 2.827 ± 0.168 | 0 | 0 |
| 13.5 | 5.500 ± 0.418 | 2.927 ± 0.143 | 2.827 ± 0.356 | 0 | 0 |
| 14 | 5.533 ± 0.528 | 2.767 ± 0.086 | 2.850 ± 0.329 | 0 | 0 |

^a^ The value is expressed as mean ± standard deviation (n = 3).

**Table S6** Changes in the concentrations of *E. coli* in raw milk and the milks treated by CHC, LTLT, HTST, and UHT stored at 5 ℃ for 14 days.

| **Storage period (d)** | **Raw milk** | **CHC** | **LTLT** | **HTST** | **UHT** |
| --- | --- | --- | --- | --- | --- |
| 0 | 2.303 ± 0.422^a^ | 0 | 0 | 0 | 0 |
| 1 | 2.623 ± 0.252 | 0 | 0 | 0 | 0 |
| 2 | 2.860 ± 0.153 | 0 | 0 | 0 | 0 |
| 2.5 | 3.987 ± 0.706 | 0 | 0 | 0 | 0 |
| 3 | 3.997 ± 0.172 | 0 | 0 | 0 | 0 |
| 3.5 | 4.467 ± 0.374 | 0 | 0 | 0 | 0 |
| 4 | 4.870 ± 0.261 | 0 | 0 | 0 | 0 |
| 4.5 | 4.850 ± 0.627 | 0 | 0 | 0 | 0 |
| 5 | 4.803 ± 0.240 | 0 | 0 | 0 | 0 |
| 5.5 | 4.343 ± 0.615 | 0 | 0 | 0 | 0 |
| 6 | 4.443 ± 0.278 | 0 | 0 | 0 | 0 |
| 6.5 | 4.893 ± 0.232 | 0 | 0 | 0 | 0 |
| 7 | 4.920 ± 0.370 | 0 | 0 | 0 | 0 |
| 7.5 | 4.953 ± 0.060 | 0 | 0 | 0 | 0 |
| 8 | 4.923 ± 0.092 | 0 | 0 | 0 | 0 |
| 8.5 | 4.920 ± 0.354 | 0 | 0 | 0 | 0 |
| 9 | 4.950 ± 0.405 | 0 | 0 | 0 | 0 |
| 9.5 | 5.020 ± 0.062 | 0 | 0 | 0 | 0 |
| 10 | 4.953 ± 0.319 | 0 | 0 | 0 | 0 |
| 10.5 | 5.043 ± 0.116 | 0 | 0 | 0 | 0 |
| 11 | 5.080 ± 0.071 | 0 | 0 | 0 | 0 |
| 11.5 | 5.117 ± 0.103 | 0 | 0 | 0 | 0 |
| 12 | 5.060 ± 0.086 | 0 | 0 | 0 | 0 |
| 12.5 | 5.110 ± 0.337 | 0 | 0 | 0 | 0 |
| 13 | 5.160 ± 0.177 | 0 | 0 | 0 | 0 |
| 13.5 | 5.207 ± 0.192 | 0 | 0 | 0 | 0 |
| 14 | 5.183 ± 0.194 | 0 | 0 | 0 | 0 |

^a^ The value is expressed as mean ± standard deviation (n = 3).

**Table S7** Changes in the pH values of raw milk and the milks treated by CHC, LTLT, HTST, and UHT stored at 5 ℃ for 14 days.

| **Storage period (d)** | **Raw milk** | **CHC** | **LTLT** | **HTST** | **UHT** |
| --- | --- | --- | --- | --- | --- |
| 0 | 6.773 ± 0.062^a^ | 6.750 ± 0.070 | 6.707 ± 0.047 | 6.620 ± 0.113 | 6.717 ± 0.108 |
| 1 | 6.683 ± 0.017 | 6.750 ± 0.041 | 6.700 ± 0.041 | 6.607 ± 0.040 | 6.720 ± 0.049 |
| 2 | 6.650 ± 0.147 | 6.727 ± 0.019 | 6.683 ± 0.118 | 6.600 ± 0.008 | 6.683 ± 0.017 |
| 2.5 | 6.607 ± 0.009 | 6.723 ± 0.054 | 6.630 ± 0.022 | 6.610 ± 0.062 | 6.690 ± 0.073 |
| 3 | 6.597 ± 0.052 | 6.693 ± 0.041 | 6.577 ± 0.205 | 6.600 ± 0.049 | 6.680 ± 0.079 |
| 3.5 | 6.563 ± 0.019 | 6.680 ± 0.022 | 6.557 ± 0.192 | 6.593 ± 0.037 | 6.687 ± 0.084 |
| 4 | 6.550 ± 0.064 | 6.640 ± 0.036 | 6.557 ± 0.086 | 6.600 ± 0.045 | 6.693 ± 0.009 |
| 4.5 | 6.540 ± 0.100 | 6.603 ± 0.005 | 6.567 ± 0.025 | 6.583 ± 0.009 | 6.670 ± 0.057 |
| 5 | 6.537 ± 0.087 | 6.613 ± 0.029 | 6.550 ± 0.000 | 6.600 ± 0.091 | 6.687 ± 0.040 |
| 5.5 | 6.483 ± 0.087 | 6.590 ± 0.028 | 6.537 ± 0.021 | 6.590 ± 0.147 | 6.657 ± 0.012 |
| 6 | 6.393 ± 0.041 | 6.547 ± 0.041 | 6.447 ± 0.037 | 6.613 ± 0.266 | 6.687 ± 0.076 |
| 6.5 | 6.380 ± 0.085 | 6.510 ± 0.054 | 6.450 ± 0.100 | 6.587 ± 0.176 | 6.683 ± 0.024 |
| 7 | 6.370 ± 0.246 | 6.470 ± 0.029 | 6.433 ± 0.103 | 6.597 ± 0.113 | 6.660 ± 0.008 |
| 7.5 | 6.427 ± 0.058 | 6.470 ± 0.029 | 6.447 ± 0.078 | 6.590 ± 0.070 | 6.667 ± 0.098 |
| 8 | 6.407 ± 0.311 | 6.457 ± 0.060 | 6.470 ± 0.050 | 6.600 ± 0.141 | 6.663 ± 0.019 |
| 8.5 | 6.380 ± 0.167 | 6.453 ± 0.031 | 6.420 ± 0.180 | 6.513 ± 0.005 | 6.667 ± 0.038 |
| 9 | 6.367 ± 0.295 | 6.457 ± 0.054 | 6.407 ± 0.048 | 6.577 ± 0.033 | 6.663 ± 0.095 |
| 9.5 | 6.360 ± 0.177 | 6.450 ± 0.037 | 6.427 ± 0.012 | 6.583 ± 0.025 | 6.650 ± 0.036 |
| 10 | 6.370 ± 0.212 | 6.457 ± 0.025 | 6.400 ± 0.113 | 6.570 ± 0.150 | 6.653 ± 0.021 |
| 10.5 | 6.377 ± 0.153 | 6.447 ± 0.041 | 6.487 ± 0.042 | 6.560 ± 0.043 | 6.657 ± 0.034 |
| 11 | 6.363 ± 0.203 | 6.443 ± 0.078 | 6.400 ± 0.082 | 6.573 ± 0.017 | 6.653 ± 0.038 |
| 11.5 | 6.347 ± 0.087 | 6.433 ± 0.009 | 6.380 ± 0.206 | 6.560 ± 0.028 | 6.637 ± 0.061 |
| 12 | 6.340 ± 0.180 | 6.443 ± 0.005 | 6.373 ± 0.205 | 6.553 ± 0.034 | 6.650 ± 0.108 |
| 12.5 | 6.350 ± 0.147 | 6.420 ± 0.024 | 6.377 ± 0.225 | 6.560 ± 0.037 | 6.643 ± 0.080 |
| 13 | 6.343 ± 0.107 | 6.417 ± 0.143 | 6.357 ± 0.186 | 6.540 ± 0.033 | 6.633 ± 0.066 |
| 13.5 | 6.320 ± 0.298 | 6.413 ± 0.012 | 6.350 ± 0.142 | 6.530 ± 0.200 | 6.627 ± 0.066 |
| 14 | 6.310 ± 0.024 | 6.397 ± 0.045 | 6.340 ± 0.086 | 6.537 ± 0.128 | 6.637 ± 0.069 |

^a^ The value is expressed as mean ± standard deviation (n = 3).

**Table S8** Changes in the acidity values (%) of raw milk and the milks treated by CHC, LTLT, HTST, and UHT stored at 5 ℃ for 14 days.

| **Storage period (d)** | **Raw milk** | **CHC** | **LTLT** | **HTST** | **UHT** |
| --- | --- | --- | --- | --- | --- |
| 0 | 0.130 ± 0.014^a^ | 0.140 ± 0.008 | 0.130 ± 0.004 | 0.150 ± 0.008 | 0.123 ± 0.005 |
| 1 | 0.140 ± 0.008 | 0.150 ± 0.008 | 0.133 ± 0.012 | 0.143 ± 0.012 | 0.133 ± 0.012 |
| 2 | 0.150 ± 0.008 | 0.150 ± 0.008 | 0.144 ± 0.012 | 0.143 ± 0.005 | 0.160 ± 0.008 |
| 2.5 | 0.143± 0.021 | 0.143 ± 0.005 | 0.153 ± 0.012 | 0.153 ± 0.005 | 0.143 ± 0.005 |
| 3 | 0.150 ± 0.016 | 0.143 ± 0.005 | 0.160 ± 0.008 | 0.143 ± 0.005 | 0.150 ± 0.008 |
| 3.5 | 0.163 ± 0.005 | 0.150 ± 0.008 | 0.130 ± 0.000 | 0.140 ± 0.000 | 0.160 ± 0.008 |
| 4 | 0.180 ± 0.008 | 0.153 ± 0.005 | 0.147 ± 0.009 | 0.140 ± 0.008 | 0.153 ± 0.009 |
| 4.5 | 0.187 ± 0.012 | 0.150 ± 0.008 | 0.143 ± 0.012 | 0.147 ± 0.005 | 0.150 ± 0.008 |
| 5 | 0.213 ± 0.009 | 0.150 ± 0.008 | 0.143 ± 0.005 | 0.150 ± 0.008 | 0.150 ± 0.008 |
| 5.5 | 0.210 ± 0.050 | 0.163 ± 0.005 | 0.160 ± 0.000 | 0.143 ± 0.012 | 0.160 ± 0.000 |
| 6 | 0.227 ± 0.017 | 0.153 ± 0.005 | 0.153 ± 0.005 | 0.150 ± 0.016 | 0.150 ± 0.008 |
| 6.5 | 0.243 ± 0.025 | 0.143 ± 0.012 | 0.160 ± 0.000 | 0.153 ± 0.012 | 0.160 ± 0.008 |
| 7 | 0.243 ± 0.021 | 0.143 ± 0.009 | 0.140 ± 0.000 | 0.150 ± 0.000 | 0.143 ± 0.005 |
| 7.5 | 0.257 ± 0.005 | 0.173 ± 0.005 | 0.183 ± 0.005 | 0.150 ± 0.022 | 0.143 ± 0.005 |
| 8 | 0.267 ± 0.012 | 0.163 ± 0.005 | 0.170 ± 0.000 | 0.163 ± 0.005 | 0.153 ± 0.005 |
| 8.5 | 0.263 ± 0.017 | 0.163 ± 0.017 | 0.173 ± 0.012 | 0.153 ± 0.012 | 0.143 ± 0.005 |
| 9 | 0.280 ± 0.033 | 0.163 ± 0.021 | 0.177 ± 0.005 | 0.163 ± 0.017 | 0.153 ± 0.005 |
| 9.5 | 0.307 ± 0.042 | 0.173 ± 0.009 | 0.173 ± 0.012 | 0.153 ± 0.009 | 0.153 ± 0.009 |
| 10 | 0.330 ± 0.014 | 0.153 ± 0.005 | 0.180 ± 0.000 | 0.173 ± 0.005 | 0.160 ± 0.000 |
| 10.5 | 0.347 ± 0.029 | 0.163 ± 0.005 | 0.167 ± 0.005 | 0.170 ± 0.000 | 0.163 ± 0.005 |
| 11 | 0.343 ± 0.029 | 0.173 ± 0.005 | 0.180 ± 0.008 | 0.180 ± 0.000 | 0.153 ± 0.005 |
| 11.5 | 0.373 ± 0.025 | 0.167 ± 0.009 | 0.183 ± 0.005 | 0.153 ± 0.021 | 0.170 ± 0.008 |
| 12 | 0.417 ± 0.009 | 0.180 ± 0.008 | 0.180 ± 0.008 | 0.160 ± 0.008 | 0.170 ± 0.014 |
| 12.5 | 0.473 ± 0.029 | 0.180 ± 0.000 | 0.173 ± 0.005 | 0.160 ± 0.008 | 0.160 ± 0.000 |
| 13 | 0.510 ± 0.049 | 0.163 ± 0.005 | 0.183 ± 0.005 | 0.173 ± 0.005 | 0.163 ± 0.012 |
| 13.5 | 0.477 ± 0.029 | 0.177 ± 0.005 | 0.163 ± 0.017 | 0.180 ± 0.008 | 0.153 ± 0.009 |
| 14 | 0.523 ± 0.009 | 0.173 ± 0.005 | 0.183 ± 0.005 | 0.163 ± 0.005 | 0.163 ± 0.005 |

^a^ The value is expressed as mean ± standard deviation (n = 3).
